# Longitudinal changes in frontopolar cortex activity during postural tasks in collegiate ice hockey athletes

**DOI:** 10.1117/1.NPh.12.3.035017

**Published:** 2025-09-30

**Authors:** Samuel R. Zeff, Douglas N. Martini

**Affiliations:** a California State University, Long Beach, Department of Kinesiology, Long Beach, California, United States; b University of Massachusetts, Amherst, Department of Kinesiology, Amherst, Massachusetts, United States

**Keywords:** repetitive head impact exposure, functional near infrared spectroscopy, dual-tasking, frontopolar cortex, quiet standing

## Abstract

**Significance:**

The effects of repetitive head impacts through contact sport participation remain poorly understood. Longitudinal assessments of their influence on neurophysiological and cognitive–motor changes can provide insights into brain function and athlete health.

**Aim:**

We aimed to quantify the effects of repetitive head impact exposure from a season of ice hockey participation on cortical neurophysiology during single- and dual-task standing balance tasks.

**Approach:**

We compared frontopolar oxyhemoglobin concentration changes longitudinally during standing balance tasks using functional near-infrared spectroscopy. Similarly, we compared motor performance from postural assessments, quantified through inertial measurement units. To quantify cognitive performance, serial-7 subtraction task accuracy was recorded.

**Results:**

Following a season of contact sport, increased frontopolar cortex activity was observed during both single- and dual-task postural tasks. In addition, longitudinal increases in transverse and coronal sway velocities were observed. Despite these changes in neurophysiology and postural control, cognitive task performance (serial-7 subtraction) remained unchanged from pre- to postseason.

**Conclusions:**

Contact sport participation alters cortical neurophysiology and cognitive–motor performance, with more pronounced longitudinal changes in frontopolar prefrontal cortex activity during weight-bearing cognitive tasks.

## 1 Introduction

Ice hockey participation exposes athletes to hundreds of head impacts per season,^1^^,^^2^ which are associated with cortical neurophysiological, cognitive, and motor alterations.^3^^–^^11^ Despite a growing concern for long-term health, the motor performance consequences of repetitive head impact exposure (RHIE) experienced from contact sport participation remain poorly understood.^7^^,^^12^ RHIE is a routine part of contact sport participation (e.g., football, ice hockey, rugby, and soccer) and typically does not result in concussion symptomology.^6^^,^^13^^–^^15^ Despite the lack of self-reported symptomology, RHIE appears to elicit cortical neurophysiologic alterations, often resulting in cognitive and motor performance deficits.^5^^,^^8^^–^^11^^,^^16^^,^^17^ However, these reported outcomes have varied throughout the literature,^18^^–^^24^ requiring further investigation to broaden our understanding of the neural mechanisms responsible for cognitive–motor impairments.

Traditional neuroimaging (e.g., magnetic resonance spectroscopy) studies show that contact sport participation disrupts cortical neurophysiology.^17^^,^^25^^–^^33^ Disrupted default mode network connectivity and axonal integrity, including prefrontal cortex areas, are observed throughout a season of contact sport and can persist for at least 6 months.^26^^,^^28^^–^^33^ However, the testing environment of these studies limits the scope of behavioral assessment, which can be performed simultaneously, making it difficult to establish a direct connection to the cognitive–motor impairments.^5^^,^^8^^–^^11^^,^^34^ Emerging evidence highlights changes in frontal cortex activity during motor tasks following RHIE,^35^^,^^36^ offering a more direct insight into the neural mechanisms contributing to cognitive–motor deficits. Increased prefrontal cortex activity during motor tasks may indicate subcortical impairment, requiring recruitment of cortical resources to maintain task performance.^37^^,^^38^ The frontopolar region (Brodmann area 10) of the prefrontal cortex facilitates information processing, task switching, and working memory.^39^^–^^41^ Importantly, the frontopolar region exhibits task-dependent activation changes in response to cognitive–motor task demands,^42^ which underscores the relevance of the frontopolar region to cognitively demanding motor tasks in the RHIE population. Assessing how RHIE influences the cortical activity during simple and cognitively demanding postural tasks may provide further clarity into the heterogenous behavioral findings in the RHIE literature.

Investigations that attempt to clarify the effect that RHIE has on motor performance provide mixed results.^7^ Both acute (within 48 h of RHIE) and cumulative [result of season(s) of head impact exposure] motor control deficits are reported.^5^^,^^8^^–^^11^^,^^34^^,^^43^ Acute postural deficits are present in some athletes following lab-based exposure to head impacts, as indicated by increased sway velocity, root mean square (RMS) sway, and modified balance error scoring system errors.^8^^,^^9^^,^^34^ Cumulative RHIE through contact sport participation is also associated with increased sway area and center of pressure displacement during postural tasks.^10^^,^^11^ However, several investigations report no acute^23^ or cumulative effects of RHIE on motor performance.^18^^–^^21^^,^^24^ Accounting for cortical mechanisms associated with performance deficits during cognitive and motor tasks may help decipher the heterogeneous results in the RHIE literature.

Cognitive deficits occur immediately and persist for at least 5 months following RHIE.^17^^,^^26^^,^^44^^,^^45^ Correlations between RHIE and working memory deficits were associated with neuronal integrity following a season of contact sport.^16^ However, preseason cortical neuronal integrity and working memory performance did not differ between contact and noncontact athletes,^16^ suggesting that cognitive performance recovers between contact sport seasons. Although completing a visual working memory task, decreased activity in the superior and middle frontal gyri (Brodmann area’s 8, 9, 10, and 46) was observed in a subset of contact sport athletes exposed to a greater number of weekly head impacts.^17^^,^^26^^,^^27^ These observations suggest an association between increased RHIE and attenuated cognitive improvement specific to contact sport athletes.^46^ Mirroring the motor dysfunction, RHIE literature reports mixed results regarding cognitive deficits following RHIE.^21^ Assessing cortical neurophysiology during motor tasks with varying cognitive loads could provide another step toward clarifying the heterogenous results of the RHIE literature.

The purpose of this study was to quantify the effect RHIE from a season of ice hockey (contact sport) participation on cortical neurophysiology during single- and dual-task standing balance tasks. We hypothesized that RHIE from a season of contact sport participation would increase frontopolar prefrontal cortex activation during single- and dual-task postural tasks, with larger frontopolar prefrontal activity increases under dual-task conditions. Furthermore, we hypothesized contact sport participation would increase sway area, RMS sway area, and sway velocity during postural tasks and reduce cognitive task accuracy.

## 2 Methods

### 2.1 Participants

Seventeen male, collegiate hockey athletes enrolled in this study. Inclusion criteria included: being between 18 and 30 years of age and being a rostered member of the university hockey team. Exclusion criteria included self-reported cognitive, attentional, auditory, and/or visual processing impairments, as well as a lower extremity musculoskeletal injury histories or surgeries that would affect gait and balance control. Self-reported number of diagnosed concussions and date of the most recent concussion diagnosis were obtained during the initial screening period but did not rule out participation from this study. Approval of this research was gained from the University Institutional Review Board, and written informed consent was obtained from all participants prior to testing.

### 2.2 Protocol

Participants were asked to perform standing balance trials under single- and dual-task conditions. Each trial began with a 20 s quiet standing baseline period, during which participants stood with their feet together, hands on their hips, and eyes closed. This was followed by a 120 s task period that varied depending on condition. During single-task trials, participants continued to stand quietly in the same posture with their eyes closed for the entire 120 s trial. During dual-task trials, participants began a cognitive task immediately after the 20 s baseline period while keeping their feet together, hands on their hips, and eyes closed. Specifically, they performed a serial seven subtraction task, counting backward by sevens from a randomly selected number between 900 and 999. Their counting task performance was recorded to be assessed following collections. Single- and dual-task condition order was randomized across participants.

### 2.3 Experimental Setup

#### 2.3.1 Cortical neurophysiology

Participants were fitted with a wireless functional near-infrared spectroscopy (fNIRS) system (Dual Brite II, Artinis Medical Systems, Netherlands) using the international 10-20 anatomical positioning system.^47^ fNIRS appears sensitive to changes in frontal cortex activity following a season of contact sport in the absence of motor performance abnormalities,^35^ as well as in persistently symptomatic individuals following a concussion.^48^ Preseason data from 12 subjects were sampled at 25 Hz, whereas data from 5 subjects were sampled at 75 Hz. All postseason data were sampled at 50 Hz. Before further processing and to improve data uniformity, data were downsampled to 25 Hz in Oxysoft (version 3.2.51), as necessary. To ensure that downsampling did not influence results, paired t-tests were conducted to compare original and downsampled data, revealing no significant differences in sample rate on median oxyhemoglobin concentration changes across all tests (p=0.99).

A total of 10 optodes bilaterally covered the left and right frontopolar lobes (the lateral portion of Brodmann area 10).^39^ Specifically, four transmitters were placed 30 mm from a single receiver, secured via distance guards, creating four long-separation channels per hemisphere. Each transmitter sent continuous waves of light with wavelengths between 840 and 760 nm used to quantify the relative changes in oxygenated and deoxygenated hemoglobin, respectively. No short-separation channels were used in this study.

#### 2.3.2 Motor performance

Participants were fitted with six inertial measurement units (IMU; OPAL V2, APMD, a Clario company, Portland, Oregon, United States) on the sternum, lumbar section of spine, each wrist, and both feet to quantify motor performance. IMU data were sampled at 128 Hz. The IMU and fNIRS systems were time synchronized via the Artinis PortaSync system for post-processing purposes.

#### 2.3.3 Cognitive task performance

Participants’ verbal responses were recorded for the serial seven subtraction task. The primary outcome variables for the serial subtraction task were the number of total attempts, total errors, and percentage of correct attempts.

### 2.4 Data Processing

Concepts from the current consensus guide on fNIRS filtering and processing were implemented to ensure reliable outcome metrics were obtained.^49^ Cortical neurophysiologic data were processed using the Homer 3^50^ software program in MATLAB (Mathworks, Natick, Massachusetts, United States). First, active raw light intensity channels were excluded using the hmrR_PruneChannels function based on signal intensities and standard deviations; if the data exceeded 1e7, the signal-to-noise threshold fell below 5, and the source-detector separation range was >45  mm.^51^ Light intensity was converted to optical density by taking the logarithm of the transmitted light. Using a modified Beer–Lambert law,^52^ light intensity was then used to estimate changes in chromophore concentrations, followed by spline and wavelet corrections to account for motion artifacts.^53^^–^^56^ Motion artifacts were assessed for each independent channel using the hmrR_MotionArtifactByChannel function.^56^ Briefly, segments of 1 s in length with a signal amplitude threshold of 5 and a standard deviation threshold factor of 40 over a 1 s time window were identified as motion artifacts.^54^^,^^55^ These artifacts were interpolated by a cubic spline via the hmrR_MotionCorrectSpline function with a smoothing parameter of 0.99^56^ and corrected using the hmrR_MotionCorrectWavelet with an interquartile range of 1.5.^53^^,^^54^ A fourth-order, zero-lag, low-pass Butterworth filter with a 0.14 Hz cut-off frequency was implemented to remove physiological noise from optical density data.^50^ Optical density was then converted to hemoglobin concentrations using the hmrR_OD2Conc function with a pathlength correction factor of 6, followed by a final motion correction using hmrR_MotionCorrectCbsi.^57^ Baseline corrections were implemented, where the mean of the 20 s quiet period was subtracted from the entire 120 s motor task signal. The median was calculated across the eight long channels covering the left and right frontal lobes and averaged to create a single orbitofrontal prefrontal cortex value for each 120 s task period for both oxy- and deoxyhemoglobin concentration changes. In addition, statistical testing revealed no significant differences between the left and right hemispheres (p>0.05), supporting the appropriateness of combining the channels into a single bilateral measure. Visual inspections of data were implemented throughout each step of processing on each respective channel to ensure both motion and physiological artifacts were not present in the post-processed signals.

Postural metrics from the IMUs were processed using Mobility Lab (APDM). The primary outcome variables for postural tasks were sway area (95% ellipse rotation [radians]), transverse plane RMS sway ($m/s^{2}$), coronal plane RMS sway ($m/s^{2}$), and transverse and coronal plane mean velocity (m/s).

### 2.5 Statistical Analysis

Statistical analyses were performed in SPSS 28.0 (SPSS, IBM Corp., Armonk, New York, United States). Kolmogorov–Smirnov tests were used to check for normality, in addition to visual inspection of histograms. All postural metrics exhibited nonnormal distributions and were log-transformed to improve normality. Relative changes in oxyhemoglobin concentrations were normally distributed. Data points greater than 3 standard deviations from the mean were excluded from further analysis. Two-way repeated measures generalized linear models were fit using Pillai’s trace to compare cortical neurophysiologic and motor changes for each independent variable across time and between conditions. Mauchly’s test of sphericity was used to determine whether the assumption of sphericity was met for each independent variable. Partial eta squared ($\eta^{2}$) was calculated as a measure of effect size for all generalized linear models.^58^^,^^59^ $\eta^{2}$ was then converted to Cohen’s d using Eq. (1),^60^ and defined as small (0.2 to 0.49), moderate (0.5 to 0.79), and large (≥0.8).^58^ In a separate analysis, concussion history was used as a covariate, where only Cohen’s d (converted from $\eta^{2}$) is reported

$$d=\frac{2\surd \eta 2}{\surd (1-\eta 2)}.$$ (1)

Differences in cognitive task performance metrics were assessed with paired t-tests, with Cohen’s d effect sizes calculated. All inferential statistical tests were evaluated with an *a priori* α=0.05. Results presented as mean [standard deviation] unless otherwise noted. Standard errors were also included for clarity.

## 3 Results

### 3.1 Participants

Of the 17 enrolled participants, one sustained a concussion during the season and was removed from analysis. Six athletes self-reported concussion histories (mean number of concussions 1.83[0.75], time from study enrolment since most recent diagnosis (3.17 years [1.35]). Two athletes, both of whom had concussion histories, had postural metrics that were greater than 3 standard deviations from the mean and were excluded from further analysis for cortical neurophysiology, postural control, and cognition. A total of 14 subjects (age: 20.86[1.68] years, height: 1.85[0.05] m) were used for data analysis. A separate exploratory sub-analysis was performed on the remaining four athletes with concussion histories (age: 21.25[1.41] years, height: 1.85[0.07] m) and 10 athletes with no self-reported concussion histories (age: 20.30[1.77] years, height: 1.85[0.04] m) (see Sec. 3.5). All participants completed preseason testing in July, and postseason testing ∼1 month following the conclusion of the season (30.9[3.3] days since last contact exposure; 283.4[6.3] days between testing sessions).

### 3.2 Functional Near-Infrared Spectroscopy

During preseason testing, athletes exhibited a mean oxyhemoglobin concentration of 0.22  μmol [0.73, SE 0.17] and a mean deoxyhemoglobin concentration of −0.06 [0.18,SE0.05]  μmol during the single-task condition. In the postseason single-task condition, mean oxyhemoglobin increased to 0.50 [0.56,SE0.14]  μmol, whereas mean deoxyhemoglobin decreased to −0.23 [0.17,SE0.04]  μmol. For the dual-task condition, preseason means were 0.77 [0.18,SE0.05]  μmol for oxyhemoglobin and −0.38 [0.18,SE0.04]  μmol for deoxyhemoglobin. These values increased in the postseason to mean oxyhemoglobin of 1.79 [0.17,SE0.04]  μmol and mean deoxyhemoglobin of −0.56 [0.43,SE0.11]  μmol.

**Fig. 1:**
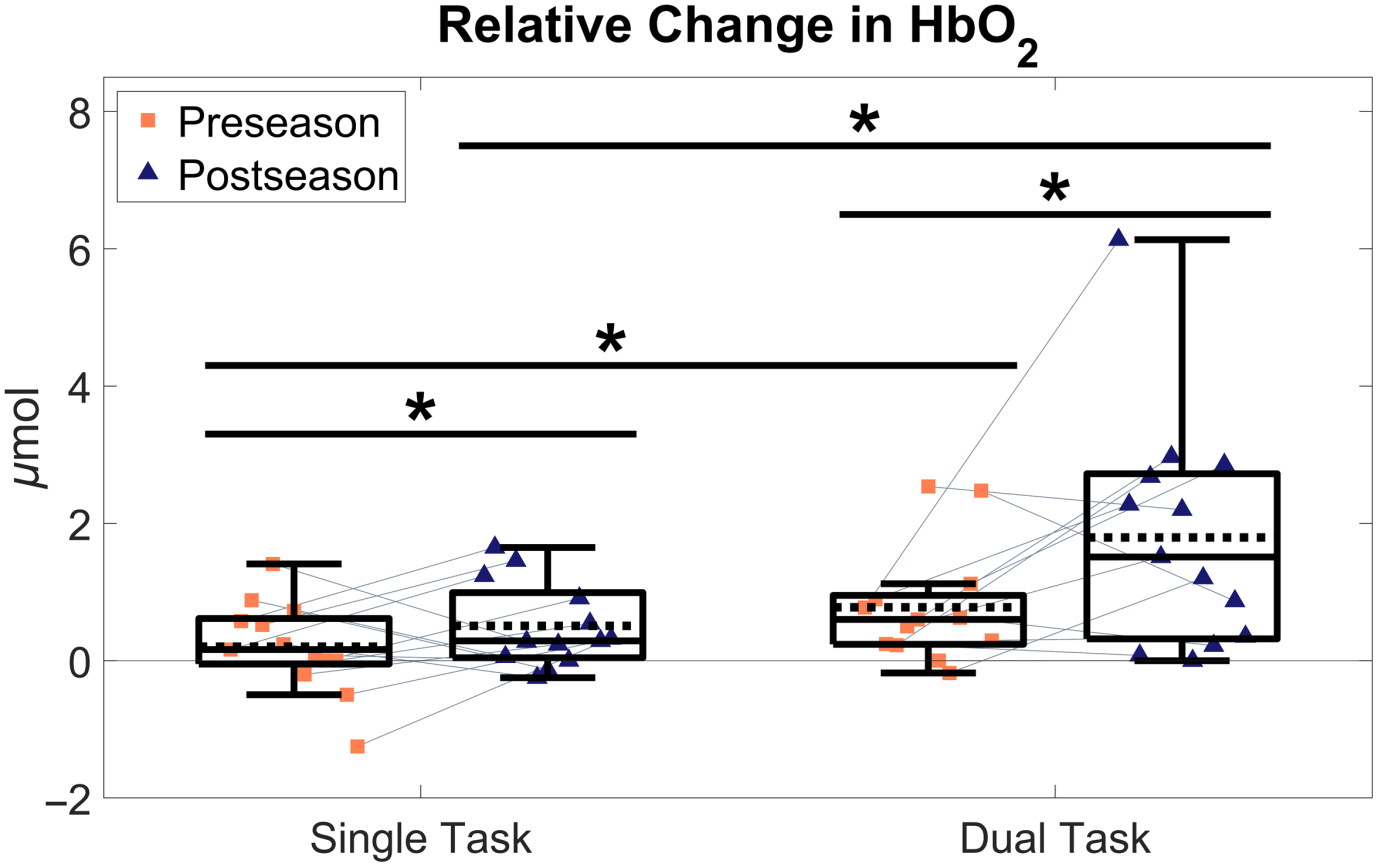
Box and scatter plots of changes in oxyhemoglobin concentrations throughout a season of contact sport. Greater values indicate a larger increase in cortical activity relative to a quiet standing period. Dashed line represents group means. Light gray lines connect participants to further highlight individual longitudinal relative changes. Completing a cognitive task significantly increased frontopolar cortex activity during both testing sessions. Separately, participating in a season of contact sport significantly increased frontopolar cortex activity during both single- and dual-task posture. Asterisks (*) indicate significant differences (p<0.05).

**Fig. 2:**
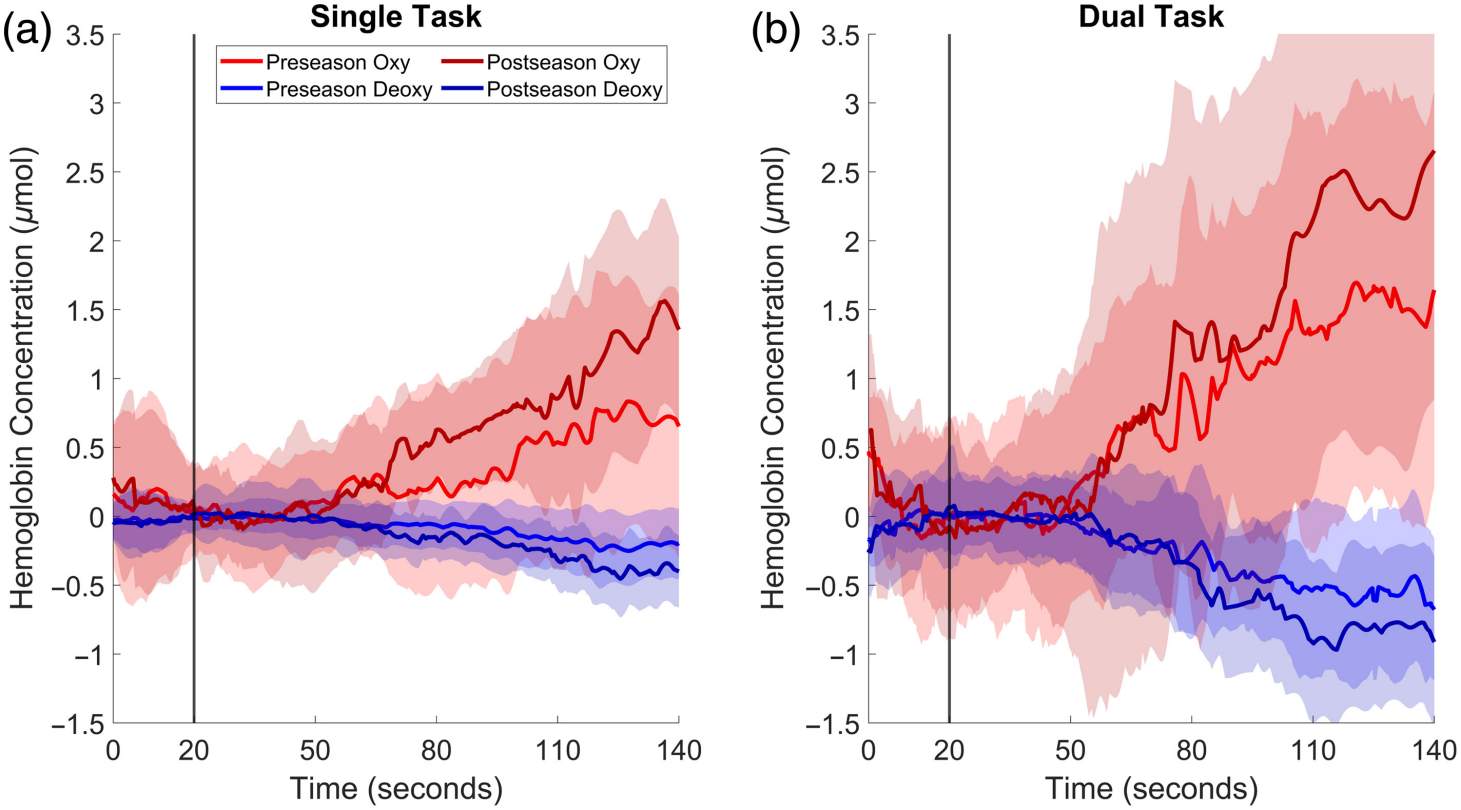
Median oxyhemoglobin and deoxyhemoglobin concentrations are shown as solid lines, with shaded areas representing individual standard deviations across all participants, segmented by time period for (a) single- and (b) dual-task conditions. The solid vertical line delineates the 20 s quiet period and 120 s task period. Participating in a season of contact sport significantly increased frontopolar cortex activity during both single- and dual-task posture.

Completing a season of collegiate varsity hockey significantly increased frontopolar cortex oxyhemoglobin concentration during postural tasks (Pillia’s $F_{\left(1,25\right)}=6.65$, p=0.016, d=1.03). Separately, the addition of a cognitive task significantly increased frontopolar cortex oxyhemoglobin concentration changes during postural tasks (Pillai’s $F_{(1,25)\;}=11.24$, p=0.003, d=1.34), but no Time X Condition interaction effect was observed despite a moderate effect size (Pillai’s $F_{(1,25)\;}=2.57$, p=0.093, d=0.64) (Figs. 1 and 2).

### 3.3 Motor Performance

A season of collegiate varsity hockey significantly increased mean velocity in the transverse (Pillai’s $F_{(1,25)\;}=11.84$, p=0.002, d=1.38) and coronal planes (Pillai’s $F_{(1,25)\;}=5.72$, p=0.03, and d=0.96) but did not significantly influence other postural metrics (Table 1). Separately, the addition of a cognitive task significantly increased RMS sway in the transverse plane only (Pillai’s $F_{(1,25)\;}=6.05$, p=0.02, d=0.98). A Time X Condition interaction effect was observed for the mean velocity in the transverse plane only (Pillai’s $F_{(1,25)\;}=5.05$, p=0.03, and d=0.90).

**Table 1:** Means ± standard deviations (standard error) for motor task variables.

	Single task		Dual task	
	Preseason	Postseason	Preseason	Postseason
95% ellipse	1.58 ± 0.34 (SE 0.01)	1.53 ± 0.30 (SE 0.08)	1.68 ± 0.38 (SE 0.10)	1.57 ± 0.10 (0.03)
RMS sway (transverse)^a^	0.14 ± 0.05 (SE 0.01)	0.14 ± 0.06 (SE 0.02)	0.18 ± 0.08 (SE 0.02)	0.19 ± 0.05 (SE 0.01)
RMS sway (coronal)	0.07 ± 0.02 (SE 0.01)	0.06 ± 0.02 (SE 0.01)	0.08 ± 0.03 (SE 0.02)	0.07 ± 0.02 (SE 0.01)
Mean velocity (transverse)^b^^,^^c^	1.00 ± 0.61 (SE 0.15)	1.06 ± 0.64 (SE 0.16)	0.94 ± 0.52 (SE 0.13)	1.79 ± 0.93 (SE 0.23)
Mean velocity (coronal)^b^	0.28 ± 0.17 (SE 0.04)	0.36 ± 0.24 (SE 0.06)	0.26 ± 0.20 (SE 0.05)	0.30 ± 0.15 (SE 0.04)

The units for each variable are as follows: 95% ellipse: radians; RMS Sway: $m/s^{2}$; velocity: m/s.

a Denotes statistically significant cognitive task effects.

b Denotes statistically significant time effects of cognitive task.

c Denotes statistically significant time * cognitive task.

### 3.4 Cognitive Performance

Cognitive task performance was assessed on 12 of the 14 athletes as two participants’ cognitive tasks recordings were not transcribable during preseason or postseason testing, resulting in the participants’ cognitive task performance being removed from further analysis. Cognitive task performance did not significantly change across time during dual-task posture. There were no significant changes in the number of errors (preseason: 2.50 [2.20, SE 0.64], postseason: 1.75 [1.17, SE 0.34]; p=0.36 and d=0.39), attempts (preseason: 28.25 [10.45, SE 3.02], postseason: 28.67 [9.92, SE 2.86]; p=0.84 and d=−0.04), or percent correct (preseason: 88.91% [11.08, SE 3.20], postseason: 92.57% [9.10, SE 2.63]) during postural tasks (p=0.38 and d=0.37). Standard deviations and standard errors are shown in brackets, respectively.

### 3.5 Concussion History

**Fig. 3:**
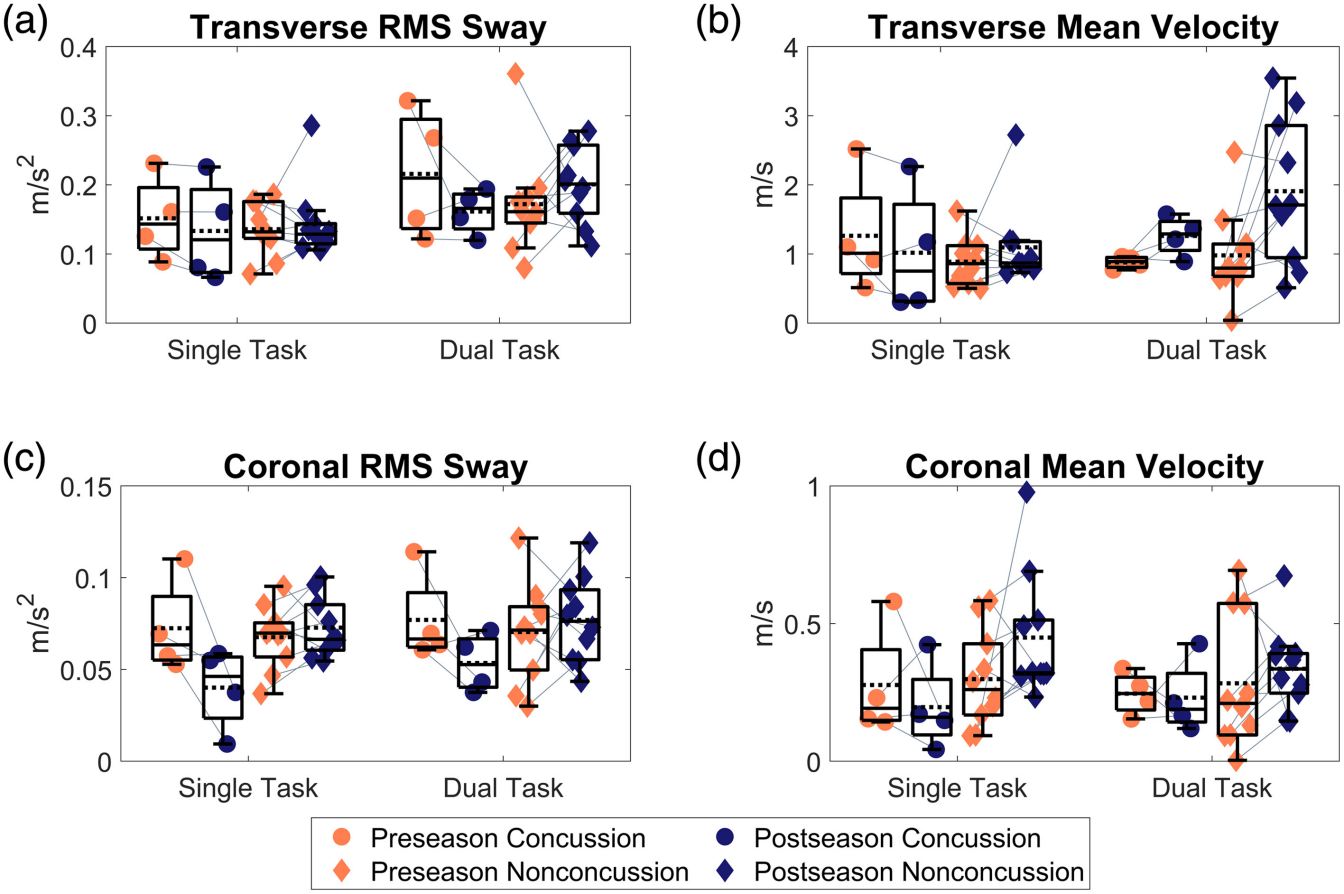
Box and scatter plots of longitudinal changes in postural metrics, including transverse (a) RMS sway and (b) mean velocity, and coronal (c) RMS sway and (d) mean velocity, separated by concussion histories. Dashed line represents group means. Light gray lines connect participants to further highlight individual longitudinal relative changes.

Small (d=0.31) Time X Concussion history effects were observed for frontopolar oxyhemoglobin concentration changes. Large Time X Concussion effects were observed for transverse RMS sway (d=1.14), coronal RMS sway (d=1.67), and coronal velocity (d=0.98; Fig. 3), whereas moderate Time X Concussion history effects were observed transverse sway velocity (d=0.78) and 95% ellipse area (d=0.54). The concussion group presents general trends distinct from the nonconcussion group in the coronal plane, with longitudinal reductions in both RMS sway and mean velocity, opposite the noncocussion group, who tended to increase across time. The transverse plane RMS sway effects may stem more from the dual-task condition, where, similar to the coronal plane, the concussion group reduced sway, whereas the nonconcussion group increased sway. Both groups displayed similar trends of transverse plane velocities across time, with limited changes during single-task conditions but longitudinal increases in mean velocities during dual-task conditions, which appear to increase to a greater extent in the nonconcussion group.

## 4 Discussion

The purpose of this study was to quantify the effect that RHIE from a season of contact sport participation has on frontopolar prefrontal cortex activity during single- and dual-task standing posture tasks. Our first hypothesis was supported, such that a contact sport season of RHIE significantly increased cortical activity during both single- and dual-task postural tasks. Our second hypothesis was partially supported, as we observed significant longitudinal increases in both transverse and coronal sway velocities following a season of collegiate ice hockey. However, no significant changes in the other postural metrics or cognitive task performance were observed. These findings highlight contact sport-related alterations in cortical neurophysiology and cognitive–motor performance, with more pronounced longitudinal changes in frontopolar prefrontal cortex activity during weight-bearing cognitive tasks. It is important to note that only the frontopolar region was examined, and assessments were limited to two time points. Therefore, conclusions regarding broader cortical changes or their temporal progression should be made with caution.

### 4.1 Cortical Activity

Our findings align with a prior report that observed increased prefrontal cortex activity following RHIE during weight-bearing tasks.^35^ Contrary to our findings and others, reductions in prefrontal cortex activity were observed during dual-task standing posture with serial-7 subtraction in nonathlete young adults.^42^ The Brodmann area frontopolar region presented herein is smaller and more inferior than the regions of interest of the prefrontal cortex in the previous studies,^35^^,^^42^ which may help explain the contrasting results. Furthermore, Mirelman et al.^42^ do not report cognitive or postural performance, limiting the ability to fully interpret the difference in observed relative hemoglobin changes. Another potential explanation for the increased frontopolar activity in hockey athletes at both timepoints might indicate a chronic effect of RHIE on frontopolar activity. The decreased prefrontal activity reported in nonathletes may reflect patterns associated with neurophysiology absent from RHIE.^42^ However, RHIE consistently increases cortical activity during motor tasks,^35^^,^^36^ and together with our findings, emphasizes it as a factor in neurophysiologic evaluations. In addition, the increases in both oxyhemoglobin and total hemoglobin concentration changes across the task duration may also be influenced by changes in arterial ${\mathrm{pCO}}_{2}$, which should be considered when interpreting hemodynamic responses.^61^ Although these differences may partially stem from competitive sport training status, the increased frontopolar activity in the present study may also reflect a compensatory mechanism to maintain cognitive–motor performance.^62^ A multiseason study with a control group is needed to confirm the observed effect from RHIE. Expanding on these previous studies, we directly related frontopolar activity to motor performance, addressing whether cortical activity patterns relate to motor performance in RHIE athletes.

### 4.2 Motor Performance

The results herein support and expand the known effects of RHIE on standing posture performance. In fact, both acute (i.e., hours) and prolonged (i.e., months and years) RHIE alter standing posture performance, suggesting that increased sway velocities may be a robust indicator of RHIE effects. Increased sway velocity, in the absence of changes in ellipse area, were found following chronic RHIE in collegiate ice hockey athletes, whereas rugby athletes demonstrated increased center of pressure sway (i.e., area) following a season of contact sport, though the duration of rest from head impacts prior to postseason assessment was not reported.^10^ A potential explanation for these reported differences could relate to the time between exposure and postseason testing. In fact, increased sway area appears to dissipate following rest from acute bouts of contact exposure.^9^ When sway velocity is reported, increases in sway velocity are observed acutely following RHIE.^8^ Combined with this acute exposure, our results expand to prolonged exposure groups, suggesting that sway velocity may be a key variable of interest in sway performance following RHIE. Future studies should longitudinally compare contact and noncontact athletes’ postural control across seasons and after the RHIE period to better understand the extent and duration of acute and chronic impairments.

### 4.3 Cognitive Performance

No significant effect of a season of contact sport exposure on the number of errors, attempts, or correct percentage of attempts was observed. Similarly, soccer athletes, considered contact sport athletes (headers), exhibited no improvement in cognitive task scores after a season of RHIE.^46^ However, noncontact table tennis and swimming athletes display longitudinal improvements in cognitive performance.^46^ RHIE appears to elicit cognitive deficits or prevent the advantages of sport participation during cognitive–motor tasks.^43^^,^^46^ Contact sport athletes demonstrate cognitive deficits during standing posture and gait tasks, compared with noncontact sport athletes.^43^ Significant serial-subtraction accuracy deficits were present in contact sport athletes during gait, without corresponding changes in other cognitive assessments.^43^ This suggests that contract sport athletes may have more issues with working memory than other cognitive domains (e.g., executive function and memory). The present findings support and expand the notion that exercise-induced cognitive improvements may be attenuated due to RHIE.

Similar to the motor performance, rest from RHIE may allow for recovery in cognitive function.^16^^,^^44^ Although contact sport career duration does not affect cognitive task accuracy during dual-task gait,^21^ it remains in question whether contact-sport-induced cognitive deficits exist acutely in the days following initial exposure when compared with noncontact athletes.^17^^,^^26^^,^^45^ Including multiple motor tasks, although longitudinally studying contact and noncontact athlete groups, will provide the evidence required to further expand our knowledge of the impact that RHIE has on motor and cognitive performance. Future studies should track cognitive task performance during motor tasks across and following a season of contact sport participation to identify potential cognitive deficits following RHIE.^43^

### 4.4 Influence of Concussion History

Additional analyses were carried out, where concussion histories were used as a covariate. Accounting for concussion history, the general trends observed present interesting findings that align with prior literature.^3^^,^^63^^,^^64^ Despite small observed effects for a longitudinal increase in frontopolar cortical activity, we did observe moderate to large effects for RMS sway and frontal and transverse plane velocities. The longitudinal dual-task changes indicate that frontal and transverse plane RMS sway, along with frontal plane velocities, tend to decrease in athletes with concussion histories but increase in the nonconcussion group. These results are consistent with observed changes in midbrain structural integrity following both concussion and RHIE.^64^ Critically, the midbrain plays an important role in postural control regulation.^65^ Combined with our exploratory results, it appears that concussion-history-specific postural control impairments may occur subcortically, at least beyond the PFC. Interestingly, concussed athletes typically experience increased RHIE (both magnitudes and frequencies) compared with athletes who avoid concussions,^66^^–^^68^ which aligns with separate reports of changes in white matter structural integrity following a season of head impact exposure.^3^^,^^63^ Though speculative, given our limited sample, persistent postural control impairment following concussion may increase midbrain susceptibility to RHIE.

### 4.5 Limitations

The present cohort consisted of division 1 male ice hockey athletes, making generalizations of these findings to other athlete groups tenuous. To quantify the influence of RHIE from contact sport, participants who were diagnosed with a concussion between testing sessions were not included in this study; although unlikely, we cannot rule out the possibility that an undiagnosed concussion occurred between testing sessions. Similarly, as participation in collegiate ice hockey exposes athletes to RHIE,^1^^,^^2^ which elicits cognitive and motor performance changes,^5^^,^^8^^,^^17^^,^^44^ we did not objectively measure head impact exposure in this cohort. In addition, the raw fNIRS data contains physiologic and motion artifacts that must be accounted for. Although we acknowledge that the use of short-separation channels is often considered best practice, access to short-separation channels was not possible in the present study. To account for this limitation, we implemented previously established signal processing methodology, which removes physiologic and motion artifacts.^49^ Formal concussion diagnosis by a medical practitioner often relies on athlete self-reporting, which can lead to underreporting and undiagnosed cases. Consequently, self-reported concussion history may be unreliable, complicating the ability to distinguish between diagnosed concussions and the cumulative effects of RHIE. Last, we acknowledge that the limited sample size in our sub-analysis prevents a thorough extrapolation of our findings.

## 5 Conclusion

We highlight distinct increases in frontopolar cortex activity following a season of contact sport participation, which appear linked to increased postural sway velocities following a season of RHIE. The findings aid in closing the knowledge gap that exists between cortical neurophysiology and cognitive–motor performance in contact sport athletes, providing a direct association between cortical neurophysiological alterations and cognitive–motor impairments. These findings contribute to the notion that contact sport participation, therefore RHIE, may have short-term implications for postural control and cognitive performance. Determining the clinical implications of these findings requires further study.

## Data Availability

Data for this paper are stored on our secure institutional cloud storage platform and are available upon request. Access must be generated upon request rather than through a public link due to institutional policy. Please contact Sam.Zeff@csulb.edu for data requests.
